# Analysis of Privacy-Enhancing Technologies in Open-Source Federated Learning Frameworks for Driver Activity Recognition

**DOI:** 10.3390/s22082983

**Published:** 2022-04-13

**Authors:** Evgenia Novikova, Dmitry Fomichov, Ivan Kholod, Evgeny Filippov

**Affiliations:** 1 Faculty of Computer Science and Technology, Saint Petersburg Electrotechnical University “LETI”, Saint Petersburg 197376, Russia; dmitry.fomichev@smartilizer.ru (D.F.); iiholod@etu.ru (I.K.); 2 Smartilizer Rus LLC, Saint Petersburg 197376, Russia

**Keywords:** privacy, federated learning, driver activity monitoring, open-source federated learning frameworks, differential privacy, homomorphic encryption, secure multi-party computations

## Abstract

Wearable devices and smartphones that are used to monitor the activity and the state of the driver collect a lot of sensitive data such as audio, video, location and even health data. The analysis and processing of such data require observing the strict legal requirements for personal data security and privacy. The federated learning (FL) computation paradigm has been proposed as a privacy-preserving computational model that allows securing the privacy of the data owner. However, it still has no formal proof of privacy guarantees, and recent research showed that the attacks targeted both the model integrity and privacy of the data owners could be performed at all stages of the FL process. This paper focuses on the analysis of the privacy-preserving techniques adopted for FL and presents a comparative review and analysis of their implementations in the open-source FL frameworks. The authors evaluated their impact on the overall training process in terms of global model accuracy, training time and network traffic generated during the training process in order to assess their applicability to driver’s state and behaviour monitoring. As the usage scenario, the authors considered the case of the driver’s activity monitoring using the data from smartphone sensors. The experiments showed that the current implementation of the privacy-preserving techniques in open-source FL frameworks limits the practical application of FL to cross-silo settings.

## 1. Introduction

To detect potentially dangerous driver activities or states such as distraction or drowsiness, modern driver monitoring systems make use of different data sources such as in-vehicle sensors, smartphones and wearable devices. Such devices collect a lot of sensitive data such as video, audio, location etc. The processing and analysis of these data are regulated by special legislative regulations such as GDPR [1] and PDPA [2], which define strict requirements to data owner’s privacy and security.

In this paper, the authors focus on the case of commercial vehicle activity monitoring based on the data from smartphone sensors. These data include readings from gyroscopes, accelerometers, GPS and other smartphone sensors, and describe the driver’s behaviour via the vehicle state. The analysis of such data contributes not only to the traffic safety and driver’s security but also to optimizing business processes as they allow enhancing simulation models for technological, construction and/or logistic processes [3]. Considering the fact that there is not much annotated data from commercial vehicles such as dumpers or excavators [4], the collaboration of the different data owners in this field is the possible solution. The recently introduced federated learning (FL) paradigm proposes a practical solution for this task. It allows decomposing machine learning processes and performing part of computations relating to data processing locally by data owners [5]. Only results of such processing are transmitted in the network to produce the aggregated analysis model [6].

Another attractive feature of the FL paradigm is a decrease in network traffic because model parameters only are transferred, and they are usually significantly smaller than the initial training data. Thus, FL proposes an attractive computational model for distributed machine learning that could be used when designing information systems such as driver monitoring systems that require processing sensitive and personal data.

However, despite these observable privacy improvements in distributed machine learning, there are no formal proofs of the privacy level guaranteed by federated learning settings [5]. Recent research on the security and privacy of the proposed FL algorithms has shown that attacks could be performed at all stages of the FL process and target all FL elements—data owners (clients), aggregating server (servers) and local and global models [5,7,8].

In this paper, the authors analyse the privacy mechanisms developed for FL systems and investigate what privacy-preserving technologies are implemented in the open-source FL frameworks such as:

- TensorFlow Federated (TFF) [9];
- Federated AI Technology Enabler (FATE) [10];
- Paddle Federated Learning (PFL) [11];
- Federated Learning and Differential Privacy (FL & DP) [12];
- FEDn [13].

The focus of this paper is privacy-preserving mechanisms implemented in these frameworks and their impact on the overall training process, i.e., global model accuracy, training time and network traffic generated during the training process for the driver’s or vehicle state recognition task. These parameters allow assessing the applicability of FL frameworks to different FL settings that are different in resource requirements, client’s behaviour and communication topology. Though there are a number of surveys devoted to different aspects of federated learning including privacy issues, they do not assess the application of privacy-preserving techniques to practical analysis tasks. For example, Kairouz et al. [5] presented the most comprehensive survey on open problems and challenges in FL including privacy issues but they focus on theoretical aspects of the FL paradigm. In [14], the authors investigate architectures and protocols used to deploy federated learning systems and provide examples of possible applications that may benefit from the usage of the FL paradigm. In [15], the authors discuss security and privacy issues in federated learning and review possible security and privacy-aware solutions, however, the survey does not contain information about the state-of-the-art privacy mechanisms implemented in the existing FL frameworks. Other research papers analyse particular attacks on privacy and integrity of machine learning processes in federated learning settings, for example, Shokri et al. [16] studied basic membership inference attacks. In [17], the authors focused on targeted data poisoning attacks, evaluated attack longevity, the impact of malicious participant availability etc.

Thus, the main contributions of this paper are as follows:

1. analysis and systematization of the privacy-preserving mechanisms developed for the FL systems;
2. analysis of the privacy-preserving mechanisms available to the users in the open-source FL frameworks;
3. assessment of their impact on overall training performance of FL systems.

The novelty of the paper consists in that all privacy mechanisms are reviewed in terms of the properties of FL systems such as computational topology, data partition, requirements to the computational resources and ability to treat drop-outs of the clients. This allowed the authors to identify an existing challenge in adopting a privacy mechanism in de-centralized cross-device settings where clients are represented by the IoT devices. The analysis results of the privacy-preserving mechanisms implemented in the open-source federated learning frameworks will be helpful when choosing a privacy mechanism as well as the framework that implements it, considering their limitations and capabilities.

The paper is structured as follows. Section 2 reviews and systematizes privacy mechanisms proposed for FL. Section 3 outlines privacy mechanisms implemented in the open-source FL systems, while Section 4 presents a detailed analysis of the privacy mechanisms supported by FL systems with the highest readiness level—FATE [10] and PFL [11]. The paper ends with conclusions and a definition of the directions of future works.

## 2. Privacy Mechanisms for Federated Learning

The privacy in driver’s behaviour and vehicle state recognition systems based on smart device sensing includes the privacy of the information that could be extracted from sensors’ readings and the privacy of the data that describes how the driver interacts with the system and its sensing components [3,18]. E. Mantouka et al. identified that existing approaches tend to “address one specific privacy threat at a time, and none of them addresses the problem of privacy in total” [3] (p. 269). The federated learning paradigm proposes a solution that guarantees not only “immediate” privacy in terms of location, current context or person’s state, but the whole analysis process starting from data processing, analysis and model deployment. It solves the problem of privacy-preserving computations by suggesting a distributed computational paradigm in which data are processed locally by data owners. Currently, the security of the proposed federated machine learning algorithms is an active research area, and it has been already shown that attacks could be performed on all stages of the FL process, and all FL elements—clients, servers and models—could be used as attack entry points.

The driver’s behaviour recognition and monitoring systems require analysis of a wide range of personal data, from location data to health data. The threat model for the federated learning includes such attacker characteristics as type, their capabilities, goals and knowledge [19]. The attackers could be classified as insiders or outsiders based on their participation in the FL process. Thus, the insiders are usually represented by collaborating clients and aggregating servers, while the outsiders are malefactors that could gain unauthorized access to the communication channel between collaborating entities and/or their information systems. In a driver or vehicle activity recognition system the insider could be represented, for example, by a dishonest driver who wants to hide fraudulent or illegitimate activity by making manipulations with sensing devices. Cybercriminals could serve as an example of outsider attackers. They could target training data in order to extract sensitive or personal data of the driver or could corrupt the model preventing it from convergence or creating a backdoor to hide some specific activity. Obviously, the capabilities of the insider are much wider, as they could manipulate data, labels as well as model parameters and their local updates. Moreover, they could have more knowledge about the FL system, for example, they could know about features used to train the model and the learning algorithm and its parameters.

The attacks could be performed during the training phase or the inference phase.

Evasion attacks are implemented during the inference phase, and they assume crafting adversarial samples that target to deceive the system. This is the most common attack on machine learning-based systems.

The target of the attacks in the training phase is the FL model itself, and depending on the attack goal, the attacker may try to learn, influence or corrupt it. It is possible to outline two generic types of attacks implemented during the training phase, they are inference attacks and poisoning attacks. The attacker can run data or model poisoning attacks to create a backdoor for future attacks by corrupting the integrity of training data set collection or the integrity of the learning process.

Inference attacks target an individual participant’s update or an aggregate of updates from all participants, and their primary goal is the privacy of data owners participating in the FL process. The application of the machine learning techniques often involves analysis of sensitive data such as health data and financial information and it has been shown that trained analysis models may be used to reveal information about the used training dataset, and therefore, impact the data owner’s privacy [16,20,21]. Based on their target, inference attacks could be classified into five categories [22]:

- class representative attacks that allow an adversary to recreate samples like the samples of the original training dataset;
- membership inference attacks that target to determine if a given sample was used in the training process;
- property inference attacks that target to learn patterns and important properties of the training dataset such as class balance in the dataset;
- sample/label inference attacks that allow an adversary to obtain not only training samples but also labels with it;
- source membership attack [23].

**Figure 1:**
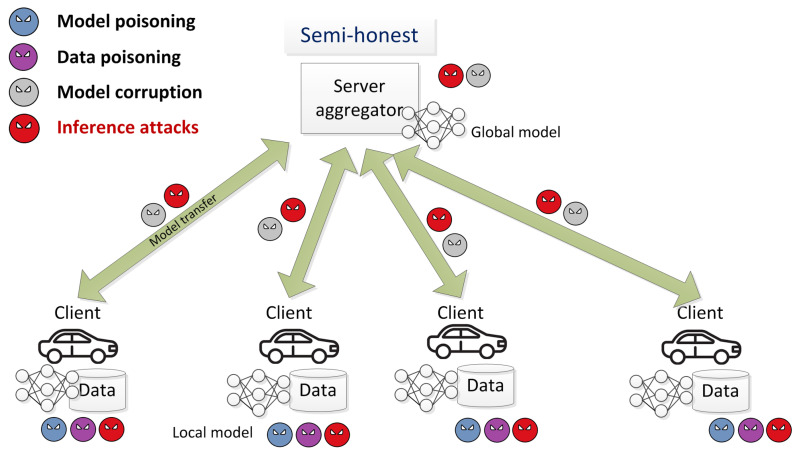
FL components and attack entry points during the training phase.

Figure 1 shows FL components and attack entry points during the training phase. The entry points of the attacks are shown by an attacker icon, while its colour is used to represent different types of the attack. The key distinction of the FL systems from traditional machine learning ones in terms of security of the training process is a wider range of the attacker’s capabilities to perform different types of attacks. This fact is explained by a larger number of participants who are actively involved in the training process. The malicious clients could not only corrupt data but model parameters as well, they could collude to perform more effective inference or backdoor attacks. The malicious server could not only corrupt the global model but also make inferences about the client’s data. In this paper, the authors focus only on inference attacks that target the privacy of the data owner.

Xu et al. suggest differentiating between the privacy of the input and privacy of computation in federated learning settings [24]. The privacy of input data relates to the protection of the inputs against leakage of information about training data, while the privacy of computation refers to the protection of data during computation as FL assumes the collaboration of many parties that could be malicious. Though the federated learning paradigm may provide a certain level of privacy, it is necessary to apply additional privacy-preserving mechanisms for both individual input and computation. Currently, the following types of privacy-preserving mechanisms are suggested:

- differential privacy (DP) mechanisms;
- data and model encryption techniques that include multi-party secure computations (MPC) and homomorphic encryption (HE);
- trusted execution environment (TEE).

**Figure 2:**
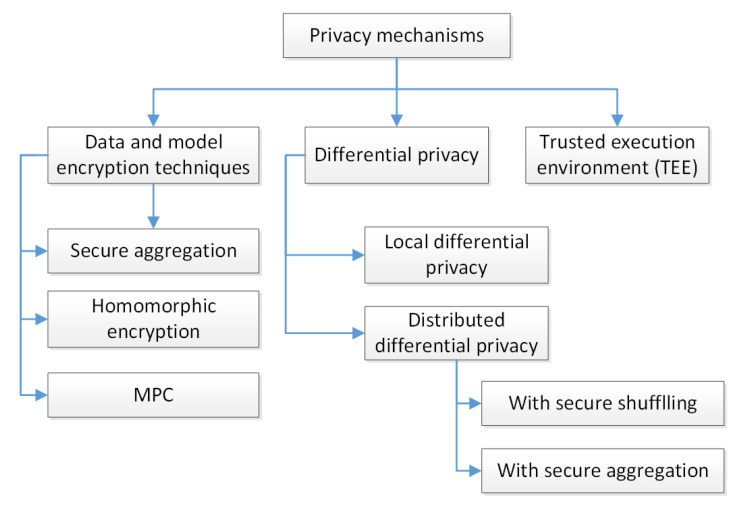
Overview of privacy-preserving mechanisms adopted for FL.

Figure 2 gives an overview of privacy-preserving approaches adopted for FL. These mechanisms are reviewed in detail in the subsections below. When analysing them, the authors analysed their ability to tolerate client dropouts and the requirements of computational resources.

The authors also considered a security model that is supported by different privacy-preserving approaches. The security model includes a type of adversary that is used to evaluate the protocol and number of clients that could be corrupted. There are two types of adversaries:

- a semi-honest adversary that could corrupt parties but follows the protocol as specified, such adversaries are also referred to as honest-but-curious);
- a malicious adversary that can arbitrarily deviate from the protocol specification, such adversaries are often called active ones.

The ability of the protocol to operate in malicious adversary settings provides stricter security guarantees than in the semi-honest adversary model. For some protocols, the number of clients that could be corrupted is an essential security parameter. If such a number is greater than a half of collaborating clients then such settings are referred to as malicious majority, the rest of the cases are referred to as honest majority.

### 2.1. Differential Privacy (DP)

Differential privacy (DP) is a formal privacy-preserving model with mathematical proof [25]. The idea of DP is to mask the input data of a particular data owner by adding random noise to them.

Differentially private algorithms are characterized by two parameters: ϵ and δ. Parameter ϵ is a quantitative privacy metric, usually denoted as privacy loss or privacy budget. It lies in the range [0;∞), the smaller value of ϵ corresponds to more secure private data. Parameter δ defines a probability that privacy loss ϵ will be exceeded. If δ=0, the differentially private algorithm provides stricter privacy guarantees.

The application of the DP mechanisms to secure data privacy requires the adoption of a particular machine learning algorithm. Gong et al. [26] showed that currently there are DP variants for almost all types of the analysis models—clustering, shallow and deep classification models.

Kariouz et al. [5] outline two models of DP in FL settings: local and distributed ones. The local model of differential privacy assumes that random noise is added locally before parameters of the local model are sent to the aggregating server. This approach is widely used by large companies such as Google and Apple when collecting statistics on device activity [27,28]. A number of research papers study privacy issues of the local DP model. For example, in [29], the authors suggested an approach that allows participants to set privacy budget ϵ locally based on their requirements. Shokri et al. [30] suggested the distributed differentially private stochastic gradient descent (DP-SGD) algorithm with a selective gradient update procedure that depends on gradient values and some given threshold. However, the local DP model suffers from critical practical issues that relate to the choice of privacy budget ϵ, as the magnitude of noise has to be chosen in consideration of the magnitude of parameters’ values across datasets belonging to all data owners requiring thus additional collaboration between clients or usage of a trusted server that may result in privacy leakage [5].

The distributed DP model is aimed to solve these problems by introducing encoding and/or shuffling techniques that add additional privacy guarantees to locally differentially private inputs of the clients. Most of them [31,32,33] are based on the encode, shuffle, and analyse (ESA) model proposed by Bitau et al. [34] that guarantees the privacy through anonymization. For example, in [31], the authors present Federated Learning in the Shuffle Model (FLAME) which is based on the ESA model and uses subsampling of input data to amplify privacy guarantees. In [33], the subsampling procedure is applied not only to data but also to clients, the authors show that this allows constructing communication-efficient FL protocols based on stochastic gradient descent with the same privacy and optimization performance level as in other recently proposed approaches [32,35].

For completeness, it is necessary to remark that differentially private algorithms are elaborated primarily for the horizontal data partition. This conclusion follows directly from the definition of differential privacy that operates with datasets differing in the record but not in the set of attributes. Currently, there is not much research devoted to the differentially private algorithms for vertically partitioned data. Existing solutions use either a semi-trusted party that assists in generating synthetic integrated databases, like in [36] or combine differentially private mechanisms and encryption techniques [37].

### 2.2. Model and Data Encryption Techniques

Secure computations is a set of approaches and technologies that aim to provide opportunities to preserve the privacy of input data when computing some function jointly. Currently, there are three different cryptography-based approaches to secure sensitive data processing:

- multi-party computation (MPC);
- homomorphic encryption (HE);
- functional encryption.

All these three approaches have different properties and usage scenarios, but all of them are applicable to the federated machine learning paradigm. For example, in MPC, two or more parties jointly compute some function *F* depending on their inputs without revealing their secret inputs. The key idea of HE is to perform computations on encrypted data. Functional encryption is a public-key cryptosystem that allows parties to encrypt their data, meanwhile, an external entity can compute a specific function on the ciphertext without learning anything additional from the underlying plaintext data [24]. The latter is achieved by the generation of the special secret key. Though there are few solutions to secure computations based on functional encryption even for federated machine learning, they are still proof-of-concept [24].

Application of the MPC protocols in FL settings assumes usage of the server-aided scheme with several servers. The clients who own data provide their data (encrypted or masked) to a set of servers to perform computations. Current implementations of the MPC protocols that could be used in real-world applications are limited in the number of collaborating computational servers—two [38], three [39] or four [40,41]. MPC protocols preserve the privacy of computations, but not of the outputs. This means that nothing will be revealed during the computations; however, the output of the function being computed may reveal sensitive information.

The key distinctive feature of the HE is that it allows the third party (e.g., cloud, service provider) to apply certain computable functions on the encrypted data while preserving the features of the function and format of the encrypted data.

In the FL setting, the application of HE limits the amount of information that other participants or aggregating servers can infer from observing the training process and model parameters.

Similarly to the MPC mechanisms, HE algorithms need to be adapted to machine learning models, aggregating function [42,43,44] and data partition [44,45]. For example, in [45], the authors presented an approach for the secure aggregation of decision tree parameters for two parties that have vertically partitioned data.

Another critical issue that needs to be considered when applying the HE protocols in FL settings is that almost all well-known schemes [46,47] allow performing computations on the data that are encrypted using one single key pair. This leads to a weaker security model because all participants have to share the same key pair, and if a client colludes with the aggregator all updates can be decrypted. For example, in [48] the authors proposed a solution that secures clients against the curious aggregating server. To do this, the clients elaborate on one joint secret key and corresponding public key and use them to encrypt model updates using an additively homomorphic encryption scheme. The aggregating server performs computations over encrypted model weights, thus having no assess to the input data. However, this approach suggests honest and non-colluding clients as, otherwise, they could easily reveal each other’s inputs. Currently, the most widely used solution to overcome these problems is based on distributed schemes of key generation [42,49].

Both MPC and HE protocols are characterized by significant computational and communicational costs, because encryption and/or decryption involve multiple modular multiplications and exponentiation operations with a large exponent and modulus (usually longer than 512 bits), making them extremely expensive to compute. Thus, the main challenge in the application of MPC and HE to secure the privacy of FL is in accelerating computation and reducing communication overhead [50,51,52]. For example, in [52], the authors suggested a software solution to reduce the encryption and communication overhead of HE-based aggregation, it uses a batch encryption technique that is based on the idea of joining gradients together as possible to form a long plaintext. There are also hardware-accelerated HE solutions that use a graphics processing unit (GPU), application-specific integrated circuit (ASIC) and field-programmable gate array (FPGA).

Nowadays, HE is often used as a building block of MPC protocols. Mixing MPC with additive homomorphic encryption allows constructing secure and computational efficient solutions [53]. Usage of HE to secure shares that computing parties are constantly exchanging allows designing computational efficient protocols for the settings with a malicious adversary and dishonest majority. The computational efficiency is achieved because authentication is done not for each exchanged share but for a secret being shared. For example, in [54] the authors presented a privacy framework for FL settings that adaptively combines different privacy-preserving approaches—fully homomorphic encryption (FHE), MPC, or secure two-party computation (STPC). Depending on the security requirements, the framework could be initialized with FHE protocol (one aggregating semi-honest server) or with MPC/STPC protocol (multiple aggregation servers). The choice of MPC/STPC depends on the possible malicious behaviour of the servers. The authors proposed to use ABY2 or MP2ML [55] for two semi-honest servers, ABY${}^{3}$ framework [39] for three semi-honest servers, the MOTION [56] framework for the majority semi-honest servers, or MP-SPDZ [53] for the majority of the malicious servers.

Thus, in general, existing encryption-based solutions, due to their performance characteristics, are practically inapplicable in cross-device settings and are typically used in cross-silo settings. They also could be used in cases when computations could be delegated to a small set of trusted computational servers that do not collude [5].

Secure aggregation (SecAgg) [57] is a lightweight MPC protocol specifically designed for the FL settings and could be used in cross-device settings. It implements secure aggregation that consists of the secure summing of the local model updates by the third party in such a manner that the third party learns only aggregated model updates. The communicational and computational costs of the SecAgg protocol strongly depend on the number of clients and the size of the data vector sent by each client. Thus, the application of secure aggregation could be inefficient when the number of clients is large.

Another factor that impacts the protocol performance is the necessity to handle clients dropouts and the potential malicious behaviour of the aggregation server. The latter problem could be solved by applying public-key cryptography. To optimize the communicational costs associated with the usage of cryptographic protocols, some recently suggested approaches use the idea of replacing complete communication graph with random subgroups of clients and application of secret sharing only for a subset of clients rather than all pairs of clients [58,59,60,61].

In [62], the authors adopt a novel secret sharing protocol FastShare, which is based on the fast Fourier transform, and propose a FastSecAgg protocol that outperforms [57] in computational efficiency. The authors show that it is robust against adaptive adversaries where the clients can adaptively be corrupted during the execution of the protocol and tolerate dropouts of up to 10% of clients.

### 2.3. Trusted Execution Environment

As defined in [63], a trusted execution environment (TEE), also referred to as a secure enclave, is “a secure, integrity-protected processing environment, consisting of memory and storage capabilities”. It is characterized by the following properties [64]:

- Confidentiality that guarantees that the current state of the code execution remains confidential unless it receives the corresponding verified notification;
- Integrity that guarantees that code alteration, as well as its execution path, could not be changed unless it receives a corresponding verified notification;
- Attestation that allows TEE to prove that code is currently executed and to demonstrate its initial state.

N. Bouacida and P. Mohapatra [65] broaden this list of the TEE properties by adding authentication that is used to verify not only code but the legitimacy of participating devices; secure communication such as communication between secure enclaves is usually protected by cryptographic protocols and private keys are stored in TEE secure environment with strict data access rights. Thus, TEE provides an opportunity to execute code remotely in a secure and private manner, making it attractive to use in FL environments when clients do not trust server-aggregators. In fact, this technology may provide privacy of the inputs and privacy of computations if both FL client and server use this technology.

Another benefit of TEE is that the code execution in a secure enclave of the untrusted device is done almost at native speed when compared to other privacy-preserving mechanisms.

In [66] the authors suggested securing layer-wise FL training on clients and aggregation on the server using TEE. They also proposed to secure only the last layer of the DNN network after training as it contains information vulnerable to membership inference attacks. To consider memory constraints of TEE, the authors include an additional step during FL training setup, when the server selects a DNN model suitable for the clients equipped with TEE.

However, the current TEE environment is available only to CPU devices and is limited in memory, thus making it impossible to take the benefits of training deep learning models on GPUs or machine learning processors. Existing research also has shown that TEEs are vulnerable to different types of attacks—attacks exploiting flows in the architectural solution of a particular secure enclave, side-channel attacks or attacks utilizing software flows such as buffer overflow and bad usage of cryptographic protocols [67,68]. Kariouz et al. [5] also outlined a problem of distribution of FL functions across secure enclaves, clients and aggregating servers.

Table 1 summarizes the properties of the reviewed privacy-preserving mechanisms with respect to properties of FL such as the ability to tolerate client dropouts and the requirements of computational resources.

## 3. Privacy-Preserving Mechanisms Implemented in Open-Source FL Frameworks

A number of FL frameworks have recently appeared. In this paper, the authors review privacy-preserving mechanisms implemented by five open-source FL frameworks that are currently under active development and demonstrate a relatively high technology readiness level. They are TFF [9], FATE [10], PFL [11], FL & DP [12] and FEDn [13].

TensorFlow Federated (TFF 0.17.0) adopts two types of privacy-preserving mechanisms. They are a secure aggregation strategy as it is presented in [57] and DP-SGD algorithm [69] customized for the federated environment.

FATE 1.5.0 focuses on the adoption of a variety of encryption-based techniques that are used to secure training of vertically and horizontally partitioned data. They implement a variety of homomorphic encryption schemes including Paillier, RSA-based and others [70]. The Diffie–Hellman key agreement protocol is used in the secure key exchange protocol.

PFL 1.1.0 supports both differentially private- and encryption-based techniques designed for training NN on horizontally partitioned data and makes use of multi-party secure computation protocol (ABY${}^{3}$ protocol [39]) to perform secure training on both vertically and horizontally partitioned data.

FL & DP 0.1.0 puts forth differentially private mechanisms to preserve the privacy of input data and implements a variety of adaptive mechanisms based on exponential, Laplacian and Gaussian noise and sensitivity based sampling and subsampling.

FEDn 0.2.0 leverages TEEs to ensure the veracity of the model updates done by clients. The technical challenge is to do this in a way that does not add unacceptable overhead in computation, since the efficiency of the model updates is also essential to federated learning.

This section reviews privacy mechanisms implemented by open-source FL frameworks. Table 2 summarizes the types of privacy-preserving mechanisms being announced and adopted by different open-source frameworks.

### 3.1. Experiment Setup

When assessing the privacy-preserving aspects of these systems the authors investigated the following aspects:

- What privacy-preserving mechanisms are implemented, and what protocols are used?
- What analysis models are they intended for?
- Are there any specific limitations on their practical application?
- How do privacy mechanisms impact the training process in terms of accuracy, time and network traffic?

As a use case, the authors considered the task of commercial vehicle monitoring. The authors studied the task of the dumpers’ activity analysis and tracking, the results of such analysis could significantly contribute to the productivity as well as the sustainability of the construction industry. Dumpers are the machines that are used to transport material such as soil, sand and rocks from the excavation point to the storage site in an earth-moving cycle. Smartphone sensors such as accelerometer and gyroscope in conjunction with the GPS could be used to monitor four main activities of the dumpers: loading the material, transporting it to the destination point, discharging it and driving empty to the excavation point to get loaded again. In this use case, the collaborating entities could be represented by the companies managing construction sites who do not want to share such information for commercial reasons and/or vehicle manufacturing companies who are interested in providing driver and/or vehicle activity monitoring systems as an additional service.

The authors used a signal data set that also describes the movement of two vehicles—dumpers of the AH and HL series. These dumpers operated at a ground remediation site in Sweden, and the collected attributes include the timestamp, speed, gyroscope and accelerometer data [71,72]. The data describe the state of the commercial vehicle—movement, whether it is idle, loading or discharging. The data set is not balanced, as it contains a log of more than five hours from the AH dumper and only a 75-min log from the HL dumper. The class distribution is also unbalanced.

The following experimental settings were used. The Google Cloud virtual machines were used as a runtime environment. Each virtual machine had the following characteristics:

- single hardware hyper-thread in 2.0–3.8 GHz core,
- RAM 6.5 GB (RW speed 24 Mb/s),
- swap: 180 GB, HDD: 30 GB (RW speed 24 Mb/s).

In the experiments, configurations with two and four virtual machines were used in order to evaluate the impact of privacy mechanisms depending on the number of clients.

To model horizontal data partition, the original data set was divided between clients into equal shares: each client stored ¼ of the original data set in the experiments with 2 and 4 clients, thus each data set contained approx. 420,000 samples with 7 attributes and 1 label.

To model vertical data partition, the authors split the data across the clients in the following way:

- four clients: the first client had three attributes, the second and third—two attributes each, and the fourth client had one attribute and one label;
- two clients: the first client had six attributes, and the second client—one attribute and one label.

The authors also reduced the size of the data set to 10,000 samples as the duration of the experiments lasted more than 4 days. The previous set of experiments [73] showed that not all FL systems mentioned above are ready for production, so in the further set of experiments the authors focus only on two FL systems—FATE 1.5.0 and PFL 1.1.0.

### 3.2. Detailed Analysis of the Privacy Mechanisms Available in FATE Framework

FATE 1.5.0 provides a quite wide variety of privacy-preserving algorithms for data analysis, they include algorithms for calculating statistics for vertically distributed data, feature selection and feature encoding. However, in this paper, the authors focus on privacy-preserving algorithms used to train models in federated mode.

Currently, FATE 1.5.0 supports the training of neural networks of different types, linear regression models and gradient boosting decision trees. Moreover, these models could be trained on both horizontally and vertically partitioned data.

To protect data privacy, FATE utilizes encryption-based techniques only—secure aggregation and different homomorphic encryption schemes. For horizontally partitioned data, FATE supports a secure aggregation strategy that uses encryption of the client’s updates and additive homomorphic encryption schemes for vertically partitioned data. Table 3 summarizes privacy-preserving techniques implemented in FATE 1.5.0.

#### 3.2.1. Secure Aggregation Strategy in FATE

This privacy mechanism is based on the algorithms proposed by the authors of [57], and is used to secure local inputs from the clients. In FATE this strategy is used to train the neural network and decision trees. In the first case, the local weights of the neural network are encrypted, in the second case, the locally computed histograms (which contain the sum of the gradient and Hessian) are encrypted.

The privacy is achieved by adding specially constructed random numbers generated on the basis of clients’ secret keys, it uses the Diffie–Hellman key exchange protocol that is implemented implicitly for the FATE end user.

**Figure 3:**
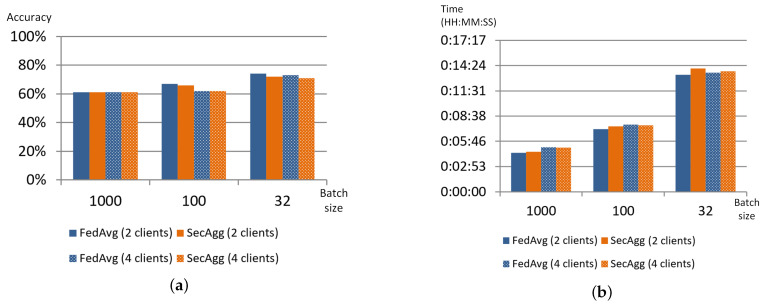

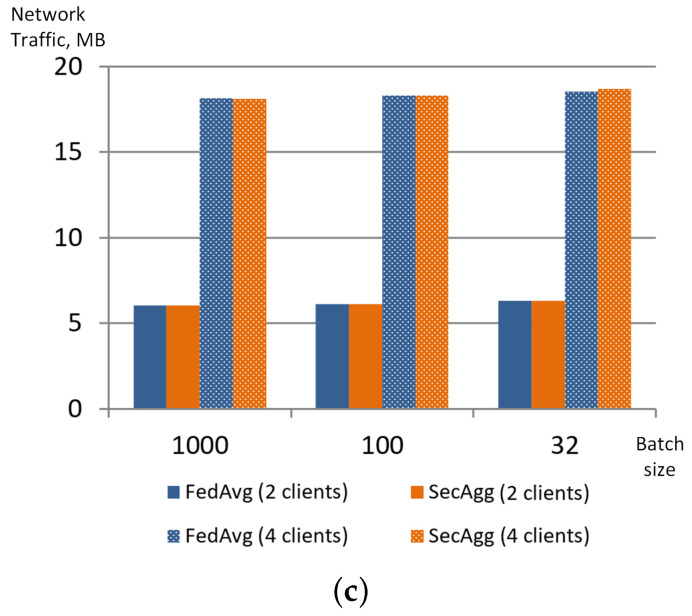
Comparison of different parameters for FedAvg and SecAgg strategies implemented in FATE: accuracy (**a**), training time (**b**), network traffic (MB) (**c**).

To assess the impact of the privacy mechanisms on the training process, the authors used FedAvg aggregation strategy [6] with similar model settings as a baseline. The results obtained showed that both strategies demonstrated very similar behaviour; their accuracy, training time and even network traffic generated by clients are almost similar. Figure 3 shows these parameters for two and four collaborating clients and the number of aggregation rounds set to 20.

Thus, the use of this privacy mechanism almost does not impact the training process. Moreover, it does not have any limitations on the number of clients participating in federated learning, it could be considered the right choice for training neural networks or decision trees.

#### 3.2.2. Homomorphic Encryption in FATE

**Figure 4:**
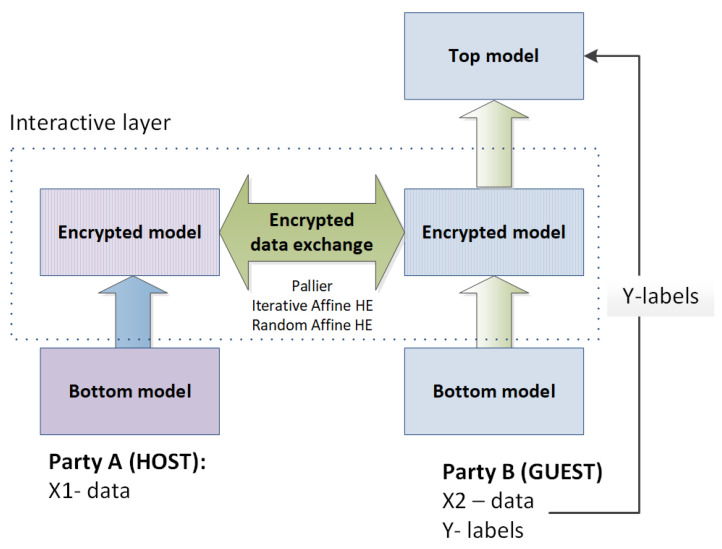
Scheme of secure NN training on vertically partitioned data implemented in the FL framework FATE.

In FATE, homomorphic encryption is used to secure the training of neural networks, decision trees and linear models for vertically partitioned data. Despite the certain differences in implementations, all the encryption-based aggregation strategies share one idea: all linear computational operations such as summing are secured by additive homomorphic encryption, while all nonlinear computations are done locally in plain text. For example, this idea is implemented in the SecureNN algorithm [76] that is used to train the neural network on vertically partitioned data. In this algorithm approach, each neuron of the network is split into linear and nonlinear components that are implemented separately on non-colluding parties. The generic architecture of the computational model is shown in Figure 4.

The authors suggest considering the bottom (unlinear) model as the “feature extraction” subtask, the interactive layer that is secured by additive HE and implements the role of the simplified classifier, and the top model fine-tunes the results of the simplified classifier. This computational model defines certain requirements for the clients (participants of computations). One client holds data and labels, and this client trains the top model (party B in Figure 4), the other clients do not have labels.

The current implementation of federated training of NN supports secure training NN with two parties only. Apart from the Paillier HE scheme, FATE 1.5.0 provides two other additive homomorphic encryption schemes—iterative affine and random iterative affine HE schemes, that differ in key generation and encryption/decryption procedures. The default encryption scheme is the Paillier HE scheme with the key length set to 1024.

Table 4 shows the results of experiments with a training NN with similar settings but on sets of different sizes and different training parameters. The accuracy does not depend on batch size but the training time grows inversely to the size of the batch. It also strongly depends on the size of the input data set. As was stated above, all experiments with vertically partitioned data were performed with a data set consisting of 10,000 records. This was done to reduce overall experiment time, as with a data set equal to 1,700,000 records the training time reaches 24 h with batch size set to 100, while for a data set containing only 10,000 records training the NN with similar settings takes 9.5 h. It should be noted that implementation of secure NN training is very resource-demanding, in terms of RAM and disc space, that is why FATE needs a lot of computational resources for installation, requiring 16 G RAM and a 500 G hard disk.

The computational model of the secure gradient boosting decision tree (SGBDT) algorithm is quite similar to the SecureNN model. There is a party that holds data and labels (referred to as active or guest) and implements secure aggregations, and parties that hold only data (passive users or host) [44]. However, opposite to the SecureNN algorithm, this model supports several passive parties. The authors detected that there is a limitation on their number when FATE 1.6.0 runs in simulation mode, the possible number of passive parties is 5. The security model is semi-honest, as the active party orchestrates the encryption process, including the key agreement procedure between active and passive parties. The encryption is applied only to secure gradients and Hessians that are computed by all parties. After receiving the feature histograms from passive parties, the active party decrypts them and finds the best gains. If the best-gain feature belongs to a passive party, the active party sends the encoded parameter back to the owner party. The current implementation of SGBDT uses the Paillier HE scheme and its modifications referred to as iterative affine and random iterative affine HE schemes.

**Figure 5:**
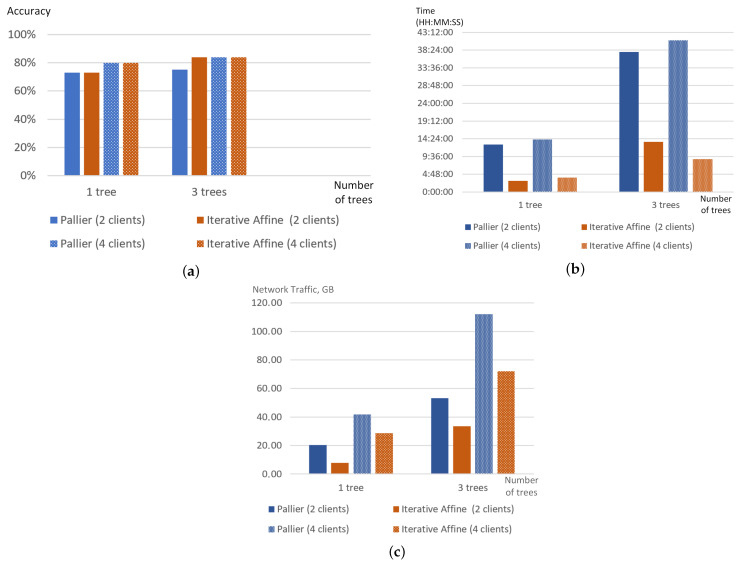
Comparison of different parameters of training process for Fast SecureBoost strategy with different initial settings in FATE: accuracy (**a**), training time (**b**), network traffic (GB) (**c**).

To save communication and encryption costs, the Fast SecureBoost algorithm is suggested and the modifications of the algorithm relate to different strategies while building decision trees. For example, in MIX mode Fast SecureBoost limits the number of trees constructed per party, layered mode supports only one guest party and one host party. Developers of FATE claim that the application of Fast SecureBoost results in a 30–50% reduction in training time. In our experiments, the authors used Fast SecureBoost and evaluated the impacts of different encryption schemes—Paillier and iterative affine HE schemes—on the accuracy, training time and network traffic. Figure 5 illustrates the results of the experiments. It is obvious that the type of encryption does not affect significantly on accuracy of models training, however, it affects considerably training time and volume of generated network traffic. Training model with Paillier HE takes an average of 3.85 times more time than using Iterative Additive HE, and network traffic are on average 1.6 times larger for Pallier HE. The number of trees also influences the training time and the network traffic. The network traffic proportionally grows with the increase in the trees’ number.

FATE 1.5.0 suggests several linear classification models implemented in the federated model for vertically partitioned data: linear regression, logistic regression and Poisson regression. These models are also secured by partially homomorphic encryption. However, the computational model slightly differs from one used in secure training of NN and SGBDT. It assumes three different roles assigned to clients—arbiter (aggregator), guest and host. Arbiter is a node (server) that orchestrates not only training processes but also key management procedures, it delivers keys to guest and host, and the guest holds data and labels, while hosts have only data. The computed gradients are locally encrypted by hosts and guests and transmitted to the arbiter, who aggregates, calculates and transfers back the final gradients to corresponding parties. To encrypt gradients, the Paillier, iterative additive and random iterative HE are used. The default encryption scheme is Paillier with a 1024 key size.

Experiments showed that, similarly to training other models secured with HE schemes, training regression models regardless of their type (linear, logistic and Poisson) is a time-consuming task. The authors evaluated training of the linear regression for two and four clients. In both cases clients held data sets of similar size, the training was performed with a batch size set to 1000. Table 5 shows the experiments’ settings and results obtained. The accuracy of the regression model trained on two clients was high, however training the model took almost 8 h, and the volume of network traffic generated by the arbiter was 11 GB. With the increase in the number of clients, the training time, as well as network traffic, increased proportionally.

### 3.3. Detailed Analysis of the Privacy Mechanisms Available in PFL Framework

Currently, PFL implements a centralized (server-aided) model for aggregating parameters of models, and almost all privacy-preserving algorithms implement a similar computational model. As currently, PFL focuses on training neural networks of different types in federated mode (regression models are also implemented as specific shallow networks with one data and one output layer) the privacy mechanisms are targeted to protect neural network parameters and use the peculiarities of neural network (NN) training.

For horizontally partitioned data, PFL supports

- DP-SGD algorithm for differentially private NN training;
- secure aggregation, encryption-based algorithm for NN training.

For vertically partitioned data, PFL implements a pair of connected protocols ABY${}^{3}$ [39] and PSI [77], where ABY${}^{3}$ is a three-party secure computational protocol, and PSI is used to find the intersection of the vertically partitioned data in a private manner.

In fact, ABY${}^{3}$ could be applied to secure processing of horizontally partitioned data, but firstly the data have to be prepared in a corresponding manner: they have to be masked and shared between the three computational parties.

Table 6 shows privacy-preserving techniques implemented in PFL 1.1.0.

To assess the impact of the privacy mechanisms on the training process, the authors used the FedAvg aggregation strategy [6] with similar model settings as a baseline.

#### 3.3.1. Secure Aggregation Strategy in PFL

Experiments showed that in general case secure aggregation (SecAgg) strategy shows results that are comparable with results obtained when training with FedAvg strategy. As in the case of FedAvg, the PFL showed unstable behaviour when training NN with batch size equal to 32 [73]. The experiments showed that the SecAgg strategy does not impact model accuracy much due to the peculiarities of the cryptographic operations. However, the authors determined interesting deviations in other parameters of the training process such as network traffic and training time.

**Figure 6:**
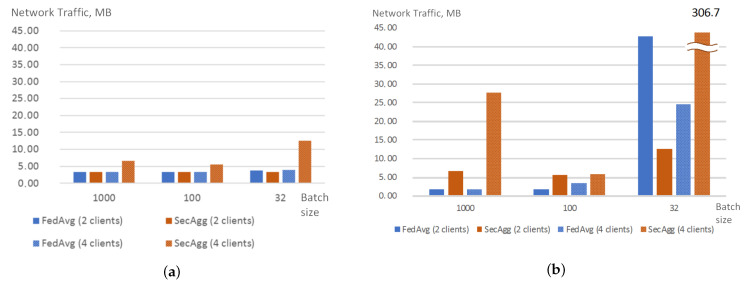
Network traffic (MB) generated by a client when training with FedAvg and SecAgg strategies for different experiment and model settings: 20 rounds (**a**), 10 rounds (**b**).

When the number of rounds is set to 20, traffic generated by SecAgg is almost the same as the traffic generated by FedAvg, and is slightly higher for the case of 4 clients and batch size set to 32. Thus, it is possible to conclude that the encryption of the model weights does not impact the overall network load. The increase of the network traffic in case of batch size set to 32 could be explained by the increase in communication between server and client to monitor the training process. When the number of rounds is set to 10, the traffic for SecAgg significantly increases. Moreover, for the case of two clients, it is possible to see that this growth depends on the size of the batch: it grows as the batch size decreases. For the case of four clients, there is no observable dependency. Figure 6 shows the averaged network traffic registered during the training process for the client.

**Figure 7:**
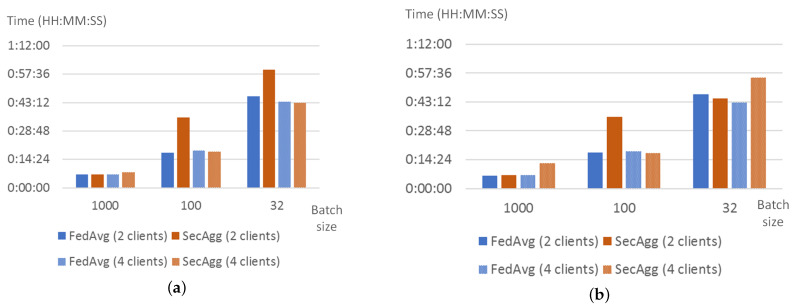
Training time of FedAvg and SecAgg for different experiment and model settings: 20 rounds (**a**), 10 rounds (**b**).

In general, the training time for both strategies is very similar, in both cases, it depends on batch size and does not significantly depend on the number of clients and the number of rounds. However, the authors observed two unusual bursts in time for batch size 100 and the number of clients equal to 2. Correlating these data with the network data (Figure 6) allows concluding that the origin of the increase in training time is not in traffic volume, however, the training time could be affected by network capacity at the moment of the experiment. Thus, this allows us to conclude that the application of weight encryption does not impact the training time much. Figure 7 shows how the training time changes when the FedAvg and SecAgg strategies are used.

#### 3.3.2. DP-SGD Strategy in PFL

PFL supports the DP-SGD strategy for training NNs and regression models. The federated implementation of DP-SGD in PFL implements the local privacy model, i.e., the data privacy protection by adding noise is done locally on the client. However, the setting of the privacy budget is done in a centralized manner, and this parameter is the same for all clients.

**Figure 8:**
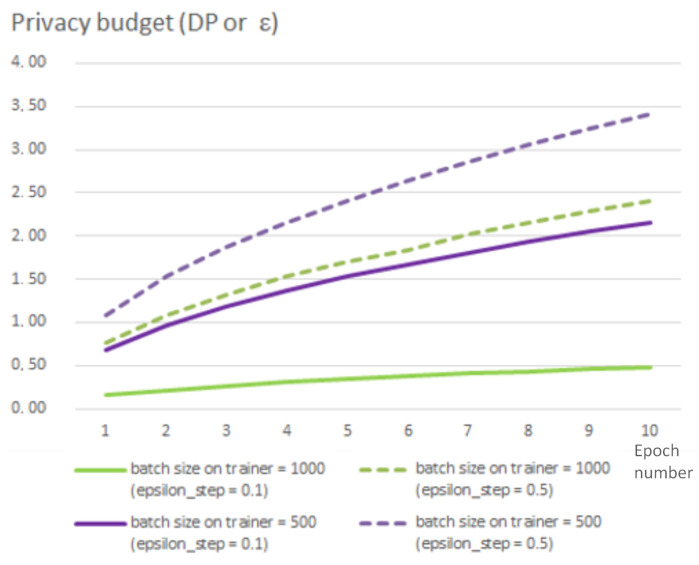
Privacy budget depending on batch size and epoch number.

It is possible to set up the privacy budget ϵ as well as the probability to break it (parameter δ). The privacy budget ϵ is set via parameter epsilon_step that is used to define the noise scale added on each step of training and is inversely proportional to the budget. Thus, the smaller values of epsilon_step correspond to the larger amount of noise added to every step of training. In the experiments, we changed this parameter in the range from 0.1 to 1.0, and these changes do not significantly impact the overall accuracy of the analysis model. It should be also noted that the privacy budget ϵ depends on the number of steps in the training process. The number of steps in their turn depends on the batch size. Thus, the privacy budget ϵ grows much quicker for smaller batch sizes than for the larger size batch. Figure 8 shows the dependence of this parameter on batch size on trainer and epsilon_step.

**Figure 9:**
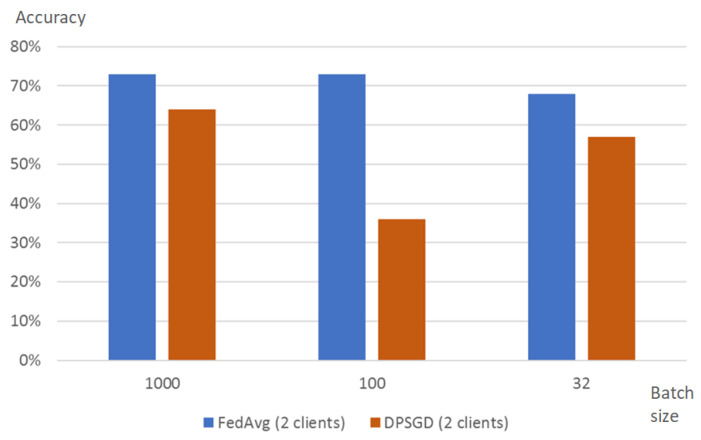
Accuracy of DPSGD strategy for two clients and round number set to 20.

The accuracy of DP-SGD turned out to be lower than the accuracy of the analysis model trained using FedAvg (Figure 9). When training with default settings, i.e., step_epsilon=0.1; δ=0.00001, the accuracy is approximately 10% lower than for FedAvg, and it falls almost twice for the case when the batch size is set to 100. The authors also defined that in contrast to the FedAvg strategy the accuracy of the DP-SGD strategy depends on the number of clients. Accuracy is 64% for the case of two clients and achieves about 75% for the case of four clients. This may be explained by the fact that the amount of data used in training grows proportionally to the number of clients: for the case of four clients, the amount of training data doubles in comparison with the case with two clients.

Unexpectedly, the training time of DP-SGD grows drastically with an increase in the number of clients and the batch size. When the authors assessed the network traffic generated by DP-SGD, it was discovered that its volume significantly exceeds traffic generated by SecAgg and FedAvg: if the traffic volume for FedAvg and SecAgg is measured in MB (Figure 6), the traffic for DP-SGD is measured in GB, and it grows almost linearly with the number of batches. At the moment, the authors explain this fact by the additional communication and computations present on each training step (as noise is added at each step).

#### 3.3.3. MPC in PFL for Vertically Partitioned Data

**Figure 10:**
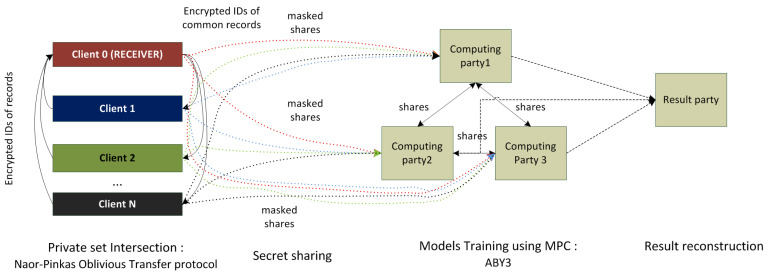
Analysis pipeline using MPC protocols implemented in the PFL framework.

PFL implements the processing of vertically partitioned data with help of MPC protocols. The generic scheme of the analysis of the vertically partitioned data is shown in Figure 10. It consists of four major steps:

(1) Definition of intersection of data sets belonging to different data owners (clients). The implementation of this step is necessary when data sets belonging to different clients do not have similar sets of records’ IDs. The clients’ data set alignment is implemented using the private set intersection (PSI) protocol, which is based on the Naor–Pinkas oblivious transfer protocol described in [77]. This protocol requires the usage of public key infrastructure, and current PSI implementation hides the details of key agreement protocol from the PFL end users, i.e., they do not need to perform it in order to set up public and secret keys in an explicit manner.
(2) Secret Sharing step. Afterwards, the clients determined the common part of their datasets, they initiate a secret sharing procedure, when they share their dataset among computational nodes. Usage of the MPC protocol as it is described above requires having two or three computational nodes that perform computations over masked data. In PFL ABY${}^{3}$ protocol is used, it considers the usage of three computational parties.
(3) Private model training process. The training of the analysis model on shared and masked data is performed by three computational nodes using the ABY${}^{3}$ protocol.
(4) Result reconstruction. After the result of a secure computation has been obtained, it is necessary to reconstruct the results, as the initial input data were split among three computational nodes. The reconstruction of the results in PFL is done based on previously used arithmetic sharing but in reverse order aggregating the shares. PFL allows setting the entity responsible for reconstructing results, this entity is called Result Party, and this role could be assigned either to the Computing party, data owner (client) or any other trusted third party.

When assessing the efficiency of MPC-based training, the authors omitted the first step (finding PSI) and the second step (making encrypted shares), only the time of the third step was assessed. Currently, in PFL, not all MPC operators for setting up neural networks are implemented. For example, PFL has batch_normalization and dropout layers, which are not implemented in MPC. That is why the test neural network was feed-forward with one input layer, two hidden layers (256 and 128 neurons), an activation layer and an output layer. Apart from this, some function activation operators and operators for calculating metrics are not implemented yet. That is why the accuracy is calculated manually as a ratio of correct predictions to all samples.

When batch size is set to large numbers the neural network does not train properly producing always the same result with accuracy ∼50%. The training time of NN using MPC depends a lot on the initial size of the dataset (i.e., number of records and attributes) and the size of the batch. Thus, for example, for a training network on a dataset consisting of 10,000 records with a batch size of 1000, the training time equals 16 min, while a training network with similar parameters on the same data set but of a larger size (1,700,000 records) results in more than 27 h. Table 7 shows the obtained results.

### 3.4. Discussion

The experiments showed that PFL and FATE implement almost similar privacy mechanisms for protecting horizontally distributed data, PFL also supports a differentially private strategy—DP-SGD—to train the NN. To protect vertically distributed data, PFL uses MPC based strategies, while FATE focuses on the application of partially homomorphic encryption. The latter has allowed FATE to support more analysis models for training them in a secured federated mode.

The secure aggregation strategy is quite similar to the FedAvg strategy in terms of accuracy, training time and network load. In PFL the authors observed bursts in traffic that are most likely explained by peculiarities of PFL internal implementation common to all strategies. The observed burst time most likely depends on network performance during experiments, and this fact has to be considered when assessing the applicability of PFL in practice. Thus, it is possible to conclude that the implementation of secure aggregation in FATE is more stable and almost similar to FedAvg.

The current implementation of DP-SGD in PFL looks rather raw, it suffers from unexpectedly high traffic as well as long training time, especially when considering that no resource consuming operations are implemented. However, it is necessary to note that accuracy grows with the number of clients using these privacy mechanisms when the federated learning environment assumes a quite large number of users.

All FATE privacy-preserving mechanisms based on partially homomorphic encryption are very time and memory-consuming and are characterized by a high volume of traffic. Searching for appropriate model parameters may result in a very long process.

Table 8 summarizes the obtained analysis results and demonstrates that the existing implementations are suitable only for the cross-silo settings with centralized communication architecture only. There are no appropriate privacy-preserving techniques for the cross-device settings. This fact is explained by their inability to handle the dropouts of the clients, and almost all of them except the secure aggregation strategy are resource-consuming. This finding reveals an urgent necessity in overcoming the gap existing between practical implementations of the privacy-preserving techniques and approaches suggested in the scientific literature (see Table 1) to make FL more useful for training models on personal and other sensitive data directly on mobile devices.

Another interesting finding is that all implemented privacy-preserving techniques support a centralized communication scheme that supposes the usage of a server-aggregator. This server-aggregator has access to data or model weights. This means that parties except party-aggregators have no possibility to make inferences about data belonging to other clients, however, the party-aggregator has such possibility, and in case of his/her compromise, the system becomes vulnerable to inference attacks. This establishes high requirements for the security and trust level of the aggregating server.

The authors’ previous research [73] has shown that the accuracy of the analysis model aimed to recognize vehicle state trained in federated learning mode is comparable to the accuracy of the analysis model trained in centralized mode when all data are collected in one data warehouse. Thus, when assessing the applicability of the federated learning and privacy-preserving techniques in particular to the task of driver’s state or vehicle activity monitoring, the crucial issue is the computational resources of the collaborating entities. In the case of commercial vehicle monitoring, the collaborating entities are represented mainly by companies or organizations that could have enough computational resources. For this case the secure aggregation strategy that is implemented both in FATE and PFL frameworks seems the most attractive as it is comparable in accuracy, time and network traffic with the FedAvg strategy that is the most commonly used aggregating strategy in FL settings. However, if the local processing is done by a vehicle itself or a smartphone, the application of such techniques is quite complicated due to high requirements for the computations resources. Another important issue to be considered when developing a driver or vehicle monitoring system based on federated learning is an aggregating party or server that all other collaborating parties trust. In practice, such aggregating party could be a state entity or a company that provides such intelligent information services.

## 4. Conclusions

Modern driver monitoring systems utilize data from different types of sensors such as in-vehicle sensors, wearable devices or smartphone sensors. These data often contain personal or other sensitive data that requires additional privacy and security protection. Federated learning is a possible practical solution that allows finding the trade-off between the privacy of the data owner and the efficiency of machine learning algorithms. It could be used to design a driver or vehicle monitoring system that enables privacy-preserving collaboration of the data owners. However, it has been shown that federated learning still has vulnerabilities that impact input data privacy.

This paper reviews and systematizes approaches proposed to enhance the privacy of the FL process. Their analysis is done with respect to the key features of FL systems such as communication topology, data partition, requirements to the computational resources and ability to treat drop-outs of the clients.

To evaluate the implemented privacy-preserving techniques in the open-source FL frameworks, the authors used the case of commercial vehicle monitoring. The experiments showed that while there are a number of research papers offering solutions that could deal with client drop-out, the privacy-preserving techniques available in the open-source FL platforms do not tolerate client drop-outs and have high requirements of computational and memory resources and network bandwidth. This allowed the authors to conclude that the current implementation of privacy-preserving techniques is suitable for the cross-silo setting only. In the context of the driver or vehicle activity monitoring task, this means that the practical application of the existing open-source FL frameworks with privacy-preserving techniques is limited to the cases when collaborating clients have enough computational resources. For example, it could be applied when designing commercial vehicle activity monitoring system, when the role of the aggregating server is implemented by trustworthy entity.

The detailed comparative analysis of the two open-source frameworks FATE and PFL in terms of privacy-preserving techniques showed that they implement slightly different approaches to enhance the privacy of the data owners, for example, FATE uses homomorphic encryption to secure computations and input data, while PFL uses MPC protocols to protect the processing of vertically partitioned data and differential private strategy to train neural networks for horizontally partitioned data. However, both of these frameworks support a secure aggregation strategy whose computational performance is comparable to the aggregation strategies without additional privacy mechanisms. In general, FATE shows a much higher technological readiness level and could be used to develop privacy-preserving FL–based driver or vehicle monitoring systems despite the high memory requirements and long training process. The recommended privacy-preserving strategy for this task is secure aggregation as it demonstrates similar results in accuracy and temporal performance as an aggregation strategy without privacy protection.

To conclude, it is necessary to note that the implemented privacy mechanisms are not secure against poisoning attacks. Malicious clients regardless of their roles in the FL system could manipulate their inputs or aggregated weights in order to bias the global model. The future direction of the research includes the assessment of the impact of the poisoning attacks on driver and vehicle activity recognition systems based on federated learning.

## Figures and Tables

**Table 1:** Properties of privacy mechanisms adopted to FL settings.

Privacy Mechanism	Privacy of Inputs	Privacy of Computations	Security Model	Dropouts	Support of Vertically Partitioned Data *	Accuracy	Specific Features and Requirements
Local DP	+	−	Honest-but-curious aggregating server	+	+ **	Decreases due to noise in comparison to FL models without any privacy-preserving techniques	Requires additional collaboration when setting privacy budget parameters
DP with shuffling	+	−	Honest-but-curious aggregating server	+	−	Decreases due to noise	Requires additional entity (shuffler), introduces additional costs due to extra communication rounds and cryptographic primitives for secure shuffling and inputs encoding
MPC	+	+	Honest majority Honest-but-curious computing party itemize	+	− Not applicable (all computations are performed by separate computational parties)	Comparable to the accuracy of FL models without MPC, decrease due to conversion from floating-point operations to modular computations	Requires additional computational entities that perform all trusted computations; high computational and resource requirements to computational entities.
Secure aggregation	+	−	Honest-but-curious aggregating server	+	−	Similar to the accuracy of FL models without MPC (input data are treated as binary vectors)	Time depends linear on the number of clients and on cryptographic primitives for public key infrastructure
HE	+	+	Malicious server Requires a trusted server for the generation of the encryption keys	− Not applicable (all computations are performed by separate computational parties)	+	Comparable to the accuracy of FL models without MPC, slight decrease due to conversion from floating-point operations to modular computations	Extremely high requirements for computational, memory and disc resources of collaborating entities.
TEE	+	+	Trusted server	+	− ***	Similar to the accuracy of FL models without any privacy-preserving techniques	Adds light overhead in CPU, memory, energy due to specific computational requirements; limits the choice of analytical models due to limited computational resources in TEE

*—the authors consider the case of vertically partitioned data as a more complicated and not widely used case. It usually requires adoption of additional dataset alignment techniques. **—it requires creating additional data set or application of encryption techniques. ***—this issue is not presented in the literature.

**Table 2:** Types of privacy mechanisms implemented in open-source FL systems.

Framework and Company	TFF 0.17.0 [9], Google Inc	FATE 1.5.0 [10], Webank’s AI Department	PFL 1.1.0 [11], Baidu	FL & DP 0.1.0 [12], Sherpa.AI	FEDn 0.2.0 [13], Scaleout Systems
DP	✔		✔	✔	
HE		✔			
MPC		✔	✔		
Secure aggregation	✔	✔	✔		
TEE					✔

**Table 3:** Privacy mechanisms implemented in FATE 1.5.0.

Data Partition Type	Type of Privacy Mechanism	Analysis Model	Implementation of Privacy Mechanism
Horizontal	Data encryption	NN (DNN, RNN, CNN) Gradient Boosting Trees	Secure Aggregation [57]
		Logistic Regression	Paillier [74]
Vertical	Data encryption	NN (DNN, RNN, CNN)	SecureNN, or Iterative Affine, or Affine Additive HE [75,76] based on Paillier scheme
		Gradient Boosting Trees	SecureBoost [44] based on Paillier HE scheme
		Linear regression Logistic regression Poisson regression	Paillier [74]

**Table 4:** Parameters of NN training using additive HE scheme for different settings.

Data Set Size	Batch Size	Accuracy	Training Time	Traffic, GB
10,000	Entire data set	98.04%	1:02:03	1.71
10,000	1000	97.15%	1:20:15	1.72
10,000	100	99.58%	9:30:59	1.78

**Table 5:** Linear regression model: experimental settings and results obtained.

Number of Clients	Data Set Size per Clients (Number of Records)	MSE	Traffic (GB), Arbiter	Training Time
2	425,000	0.0002	11.30	7:57:312
4	425,000	1.44	65.00	13:54:18

**Table 6:** Privacy mechanisms implemented in PFL 1.1.0.

Data Partition Type	Analysis Model	Type of Privacy Mechanism	Implementation of Privacy Mechanism
Horizontal	NN Linear regression Logistic regression	Differential privacy	DP-SGD [69]
		Data encryption	Secure Aggregation [57]
Verical	NN Linear regression Logistic regression	Data encryption	PSI [77] + ABY${}^{3}$ [39]

**Table 7:** Parameters of NN training using MPC for different settings.

Data Set Size	Batch Size	Training Time	Accuracy	Network Traffic (GB)
10,000	10,000	0:13:58	51.00%	35.80
	1000	0:16:35	53.75%	36.40
	32	2:11:39	99.00%	58.40
1,700,000	1000	27:19:22	49.67%	Not measured

**Table 8:** Properties of the privacy mechanisms implemented in FATE and PFL and mapped to the FL system properties.

Data Partition	Clients’ Settings	Communication Topology	Framework and Privacy Mechanisms	Impact on Overall Learning Process		
				Accuracy	Training Time	Network Traffic
Horizontal	Cross-silo	Centralized	FATE and Secure Aggregation	Similar to FedAvg strategy in terms of accuracy.	Similar to the FedAvg strategy.	Similar to the FedAvg strategy.
			FATE and Homomorphic encryption (Paillier encryption scheme)	Similar to FedAvg strategy.	Extremely time-consuming training strategy.	Extremely high volume of traffic.
			PFL and Secure Aggregation	Comparable to FedAvg strategy.	Comparable to FedAvg strategy.	High network traffic. There is an observable dependency between training time and network performance.
			PFL and Differencial privacy (DP-SGD)	The accuracy is lower the accuracy in FedAvg strategy, but it increases with growth of clients’ number.	High when compared with similar settings without privacy mechanisms.	The network traffic is extremely high and is measured in GB.
		De-centralized	Not supported	Not supported	Not supported	Not supported
	Cross-device	Centralized	Not supported	Not supported	Not supported	Not supported
		De-centralized	Not supported	Not supported	Not supported	Not supported
Vertical	Cross-silo	Centralized	FATE and Homomorphic encryption (SecureNN, SGBDT)	The accuracy is comparable with the training model in centralized mode.	It is a very time-consuming strategy.	It is characterized by a high volume of traffic measured in GB.
			PFL and MPC (ABY${}^{3}$)	The accuracy is comparable with training model in centralized mode, however it is significantly lower for neural networks when training on large batches.	It is a very time-consuming strategy.	It is characterized by a high volume of traffic measured in GB.
		De-centralized	Not supported	Not supported	Not supported	Not supported
	Cross-device	Centralized	Not supported	Not supported	Not supported	Not supported
		De-centralized	Not supported	Not supported	Not supported	Not supported

## Data Availability

Not applicable.

## Abbreviations

The following abbreviations are used in this manuscript:

ASIC	Application-Specific Integrated Circuit
CNN	Convolutional Neural Network
CPU	Central Processing Unit
DNN	Deep Neural Network
DP	Differential Privacy
DP-SGD	Differentially-Private Stochastic Gradient Descent
FedAvg	Federated Averaging Aggregation Function
FHE	Fully Homomorphic Encryption
FL	Federated Learning
FPGA	Field-Programmable Gate Array
GDPR	General Data Protection Regulation
GPS	Global Positioning System
GPU	Graphics Processing Unit
HE	Homomorphic Encryption
IoT	Internet of Things
MPC	Multi-Party Secure Computation
NN	Neural Network
PDPA	Personal Data Protection Act
PSI	Private Set Intersection
SecAgg	Secure Aggregation
SGD	Stochastic Gradient Descent
STPC	Secure Two-Party Computation
TEE	Trusted Execution Environment
